# Enhanced applications in dentistry through autoclave-assisted sonochemical synthesis of Pb/Ag/Cu trimetallic nanocomposites

**DOI:** 10.1016/j.ultsonch.2024.106966

**Published:** 2024-06-22

**Authors:** Kanagasabapathy Sivasubramanian, Yuvaraj Tamilselvi, Palanivel Velmurugan, Fatimah Oleyan Al-Otibi, Raedah Ibrahim Alharbi, Vinayagam Mohanavel, Sivakumar Manickam, Jeyanthi Rebecca L., Basavaraj Rudragouda Patil

**Affiliations:** a Centre for Materials Engineering and Regenerative Medicine, Bharath Institute of Higher Education and Research, Selaiyur, Chennai, Tamil Nadu 600073, India; b Department of Botany and Microbiology, College of Science, King Saud University, PO Box -2455, Riyadh 11451, Saudi Arabia; c Petroleum and Chemical Engineering, Faculty of Engineering, Universiti Teknologi Brunei, Bandar Seri Begawan BE1410, Brunei; d Department of Industrial Biotechnology, Bharath Institute of Higher Education and Research, Selaiyur, Chennai, Tamil Nadu 600073, India; e College of Pharmacy, Yeungnam University, Daehakro-280, Gyeongsan 38541, Republic of Korea

**Keywords:** Trimetal, Composite, Oral cavities, Stitches, Nanoparticle, Antibacterial, Antibiofilm

## Abstract

In recent years, researchers have increasingly focused on the development of multiphase trimetallic nanocomposites (TMNC) incorporating ternary metals or metal oxides, which hold significant potential as alternatives for combatting biofilms and bacterial infections. Enhanced oral health is ensured by the innovative techniques used to effectively prevent bacterial adherence and formation of biofilm on dental sutures. In this investigation, TMNC, which consists of Pb, Ag, and Cu, was synthesized using an autoclave-assisted sonochemical technique. Following synthesis, TMNC were characterized using FTIR, XRD, BET, XPS, TGA, and Raman spectroscopy to analyze their shape and microstructure. Subsequent evaluations, including MTT assay, antibacterial activity testing, and biofilm formation analysis, were conducted to assess the efficiency of the synthesized TMNC. Cytotoxicity and anti-human oral squamous cell carcinoma activities of TMNC were evaluated using the Human Oral Cancer cell line (KB) cell line through MTT assay, demonstrating a dose-dependent increase in anti-human oral squamous cell carcinoma activity against the KB cell line compared to the normal cell line, resulting in notably high cell viability. Furthermore, an ultrasonic probe was employed to incorporate TMNC onto dental suturing threads, with different concentrations of TMNC, ultrasonic power levels, and durations considered to determine optimal embedding conditions that result in the highest antibacterial activity. The inhibitory effects of TMNC, both in well diffusion assays and when incorporated into dental suturing threads, against gram-positive (*Staphylococcus aureus*) and gram-negative (*Escherichia coli*) bacteria on Mueller-Hinton agar (MHA) were assessed using various concentrations of TMNC. The results of the study indicated that the efficacy of TMNC in inhibiting bacterial growth on dental suturing threads remained impressive, even at low concentrations. Moreover, an evaluation of their potential to destabilize biofilms formed by *S. aureus* and *E. coli*, the two pathogens in humans, indicated that TMNC would be a promising anti-biofilm agent.

## Highlights

• Autoclave-assisted ultrasonic probe employed nanocomposites (TMNC) synthesis.
• FTIR, XRD, BET, XPS, and Raman spectroscopy to analyze their physical structure.
• Ultrasonic probe employed to incorporate TMNC onto dental suturing threads.
• Biological activity of the synthesized TMNC were evaluated.
• Cytotoxicity was evaluated using the Human Oral Cancer cell line (KB) cell line.

## 1 Introduction

The advancement of technology has significantly influenced the development of dental materials, which have undergone various stages of evolution over time. Over generations, the scientific community has been striving to discover materials with the essential qualities required for use in oral cavities. Dental surgeries often require the use of stitches or sutures to close wounds and aid in the healing process. While these sutures are essential for successful dental procedures, they can inadvertently promote the growth of bacteria and biofilms due to the abundance of microbes present in the oral cavity [1]. Microbial and biofilm issues within dental sutures can lead to complications such as infections, delayed healing, and persistent inflammation [2]. Bacterial presence on suture material poses a high infection that may delay the patient’s recovery and oral health. Additionally, biofilms, organized clusters of bacteria surrounded by a protective layer, are highly resistant to antimicrobial substances, making them challenging to eliminate and increasing the risk of infection [3]. Overcoming these challenges is crucial for ensuring successful oral surgery outcomes and minimizing potential complications. Dental professionals employ aseptic suturing techniques and guide patients regarding post-operative wound care and oral hygiene. Furthermore, researchers and medical practitioners are exploring novel methods to prevent bacterial adhesion and biofilm formation on dental sutures. Progress in antimicrobial-coated sutures and the development of novel materials such as mono, di, or tri metallic nanoparticles offer promising alternatives for deterring bacterial attachment and biofilm formation on dental sutures. These advancements have the potential to improve patient outcomes and enhance overall oral health [4]. Early identification and prompt treatment of any signs of infection or delayed healing are crucial for ensuring effective oral surgery and reducing the risk of potential complications [5]. In recent years, trimetallic nanoparticles have emerged as a promising alternative for halting biofilm formation and combating bacterial infections. These nanoparticles combine the antimicrobial properties of three distinct metals, lead (Pb), silver (Ag), and copper (Cu), resulting in a synergistic effect that enhances their antimicrobial efficacy against a wide range of microorganisms [6].

This study investigates the potential of trimetallic Cu/Ag/Pb nanoparticles as a promising solution for addressing biofilm formation and bacterial infections. Trimetallic nanoparticles are used in catalysis, biomedicine, antimicrobial, active food packaging, and sensing. Unique features distinguish trimetallic nanoparticles from monometallic and bimetallic ones. These nanoparticles can be synthesized via chemical reduction, precipitation, thermal, microwave-assisted, and other processes. Unfortunately, most of these technologies are expensive and environmentally damaging. We explored the underlying mechanisms of their antimicrobial activity, examining how they disrupt bacterial membranes, interfere with cellular processes, and inhibit biofilm formation. Furthermore, we examined the application of trimetallic nanoparticles in diverse contexts, including dental care, medical implants, and other industrial settings where complications related to biofilms are prevalent. Recognizing the potential of trimetallic Cu/Ag/Pb nanoparticles in preventing biofilm formation and managing bacterial infections presents exciting opportunities for the development of innovative and effective antimicrobial strategies. By shedding light on the latest advancements in this domain, this study aims to contribute to ongoing efforts in addressing biofilm-related challenges and improving patient outcomes across various medical and industrial sectors. When a heavy metal undergoes high-pressure treatment in the presence of water vapour within an autoclave, it can serve as a corrosive agent, especially in the presence of dissolved oxygen and other impurities [7]. Pressurized vapour weakens the metal and compromises its structural integrity, leading to the formation of metal oxides or other compounds through the corrosion process [8]. In high-pressure environments, hydrogen atoms from water molecules can penetrate the metal crystal lattice, reducing flexibility and potentially causing brittleness and susceptibility to fractures due to hydrogen embrittlement [9]. Phase transformation in specific metal crystal structures can alter mechanical and thermal properties [10]. Additionally, exposure to high-pressure water vapour can form metal hydrates and alter the metal’s surface finish, leading to etching, pitting, or other surface modifications due to chemical reactions or physical interactions [11]. Treating an aqueous metal solution with an ultrasonic probe involves exposing the solution to ultrasonic waves for specific purposes or applications. The ultrasonic probe generates high-frequency sound waves by inducing cavitation bubbles in the liquid medium [12]. The alternating pressure of ultrasonic waves causes rapid expansion and collapse of these bubbles, creating localized hot spots with high pressures and temperatures, resulting in powerful shear forces and shock waves within the solution. The extreme conditions generated during cavitation lead to various physical and chemical effects that facilitate the formation of metal nanoparticles, including nucleation, growth agglomeration, reduction, enhanced mixing, and reaction rates [13].

Furthermore, ultrasonic-assisted synthesis provides numerous advantages for the production of metal nanoparticles. These include accelerated reaction rates and reduced synthesis times, precise control over particle size and distribution, the capacity to generate nanoparticles with enhanced properties, high purity, and an energy-efficient and environmentally friendly synthesis approach [1]. This method finds applications across diverse fields, including nanotechnology, materials science, catalysis, and biomedicine. It enables researchers to fabricate a wide range of metal nanoparticles with customized properties, thereby serving as a valuable tool for advancing nanomaterials and their applications [14]. Nevertheless, it is crucial to carefully optimize the experimental conditions to achieve the desired nanoparticle characteristics and avoid unintended side reactions. The synthesis of Ag and Pb nanoparticles, as well as Cu combined metal composite, was conducted through the co-reduction of the metal precursors. Metals like Ag, Au, Cu, Zn, and Fe exhibit higher potential activity against microbes, as demonstrated in the antibacterial assay. Metal oxides, including copper oxide, zinc oxide, iron oxide, titanium oxide, manganese oxide, silicon dioxide, indium oxide, aluminium oxide, and chromium oxide, also demonstrate enhanced antimicrobial activity. Metal and metal oxide nanoparticles disrupt cell membrane binding and release metallic ions that interact with proteins and enzymes within the bacterial cell wall. These nanoparticles can target the bacterial cell wall through various mechanisms, including electrostatic attraction, Vander Waals forces, and hydrophobic interactions [15]. Different types of nanoparticles utilize distinct mechanisms to inhibit bacterial populations by forming pores on the surface of bacterial cell membranes. This leads to radical generation, formation of reactive oxygen species, inhibition of enzyme activity, disruption of proteins and DNA, modification of gene expression levels, and induction of oxidative stress. Ag nanoparticles, whether used alone or in combination with other metals or nanomaterials, possess unique chemical stability, catalytic activity, and enhanced interaction with microorganisms.

Factors such as large surface area to mass ratio, zeta potential, surface morphologies, crystal structures, like smaller and adjustable particle sizes and shapes collectively contribute to enhanced bacterial prevention by enabling close interaction with microbial membranes. Moreover, the effectiveness of nanoparticles in killing germs is also influenced by factors such as bacterial strain, environmental conditions, and duration of exposure. The prominent antibacterial properties of metal oxide nanoparticles have been examined against both Gram-positive and Gram-negative bacteria encompassing strains that are resistant to antibiotics. Similar to AgNP, AuNP too exhibit significant antibacterial activities; however, the precursors used for the synthesis can be expensive. Furthermore, metal oxide nanoparticles such as ZnO, CuO, TiO_2_, Al_2_O_3_, and Fe_2_O_3_ have demonstrated potent antibacterial effects against both Gram-positive and Gram-negative bacteria. This paper provides an overview of the synthesis, applications and challenges of trimetallic nanocomposites in cytotoxicity assays, antimicrobial activity, and catalytic activity.

## 2 Materials and methods

### 2.1 Materials

Lead nitrate (Pb(NO_3_)_2_, 99.8 %), Silver nitrate (AgNO_3_, 99.8 %), and copper nitrate (Cu(NO_3_)_2_, 99.8 %) were received from Sigma-Aldrich. Double-distilled water was utilized to prepare solutions and perform other experimental procedures. Additionally, *Escherichia coli* and *Staphylococcus aureus* strains were acquired from Sree Balaji Dental College and Hospital in Chennai, India. All chemicals were used in their as-received state without undergoing further purification.

### 2.2 Synthesis of trimetallic nanoparticles

The metal composite was synthesized using the metallic precursors silver nitrate, lead nitrate, and copper nitrate. Pb, Ag, and Cu trimetallic nanoparticles were synthesized using a novel technique, as reported earlier [16], [17]. Equal amounts of 1 % w/v Pb(NO_3_)_2_, 1 % w/v AgNO_3,_ and 1 % w/v Cu(NO_3_)_2_ were added to a conical flask containing 20.0 mL of double-distilled water (DDW). This metallic mixture was then autoclaved at 121 °C for 30 min. The high pressure and temperature inside the autoclave can facilitate the reduction of metal ions, leading to the formation of tiny crystalline particles. The resulting solution exhibited a light–dark colour, indicating that the initiation of nanoparticle formation occurred under pressure. Following the autoclave treatment, the solution underwent 15 min of microwave treatment and 30 min of sonication using a probe. Subsequently, the colour of the reaction mixture changed, and it was subjected to microwave treatment once again to reduce the concentration. This process aids in converting the solution into powder form, which was then calcinated at 900 °C for 2 h using a muffle furnace and alumina crucibles. As a result, a dark-colored Pb, Ag, and Cu trimetallic metal nanocomposite was obtained. The resulting powder was stored in a glass vial for subsequent experiments.

### 2.3 Characterization of TMNC

#### 2.3.1 FESEM/EDAX analysis

The surface morphologies of the produced TMNC were examined using a Field Emission Scanning Electron Microscope (FESEM, Zeiss SUPRA 55 Sapphire, Germany). Additionally, elemental mapping of the TMNC was performed using FESEM combined with Energy Dispersive X-ray Analysis (EDAX) using the SwiftED 3000 SDD detector [18], [19]. Surface area and porosity of the obtained TMNC were assessed using a Brunauer–Emmett–Teller (BET) analyzer (Quantachrome Nova 2000e) [20]. The thermal stability of the metal TMNC was analyzed using thermogravimetric analysis (TGA, Perkin Elmer). Approximately 10 mg of TMNC was subjected to TGA using a Mettler Toledo TGA/ SDTA851e apparatus at a heating rate of 10 °C/min, ranging from 25 to 600 °C [21]. The elemental composition and surface states of the TMNC were examined using an X-ray photoelectron spectrometer (XPS). Specifically, XPS measurements were conducted using an Omicron ESCA Oxford Instrument while exposing the TMNC to Al-Kα radiation with an energy of 1486.6 eV. The binding energies (BEs) were adjusted by scaling using the accepted value of 284.6 eV for the C1s BE, which accounts for accidental carbon contamination. The functional groups of the synthesized TMNC were identified using FTIR (Fourier-transform infrared spectroscopy). The FTIR spectrum was recorded within the range of 4000–500 cm^−1^ with a resolution of 16 cm^−1^ using a Nicolet Summit LITE FTIR spectrometer (Thermo Fisher Scientific, USA). The measurements were conducted in the attenuated total reflectance (ATR) sampling mode [22]. The crystalline and amorphous states of the samples were determined using the X-ray diffraction (XRD) technique [20]. XRD analysis was performed using a Philips powder diffractometer system, which utilized Cu Kα radiation with a wavelength of 1.54060 nm, operating at 40 kV and 30 mA. The scanning range was set from 10° to 80° with a scanning rate of 2°/min.

#### 2.3.2 SEM

The structural morphology of the TMNC embedded under the optimum conditions of dental suturing thread was analyzed using SEM [23]. SEM images were captured for both the TMNC-coated dental suturing thread before and after washing, allowing for the examination of any changes in morphology.

### 2.4 Optimization

#### 2.4.1 Optimization of embedding the TMNC on dental suturing thread using an ultrasonic probe and its antibacterial activity

The sterile dental suturing thread (Training purpose suture thread, Synthetic, Absorbable, Monofilament, Meril POLYAMIDE, Suture NYL01 3362) was obtained from an online marketplace for the purpose of embedding the TMNC onto the dental suturing thread. 0.1 g of TMNC was dissolved in 1 mL of Milli-Q® water and vortexed for 30 min to facilitate dissolution. Subsequently, ultrasonic water bath treatment was employed to ensure a uniform TMNC dispersion. The dental suturing thread was impregnated with the TMNC emulsion obtained under optimized conditions to investigate its antibacterial and antibiofilm properties. The process of optimizing incorporation included evaluating three factors.: concentration, ultrasonic power, and duration, to achieve the best results. Various concentrations (20, 40, 60, 80, 100, 120, and 140 µl/1 mL) of the dissolved TMNC (0.1 mm) were utilized for embedding onto the dental suturing thread. The TMNC sample was exposed to ultrasonic power ranging from 30 kHz to 90 kHz during the embedding process, with varying time intervals ranging from 1 to 10 min. Each parameter was evaluated for its antibacterial properties to identify the optimal coating conditions. The optimized composite-embedded dental suturing thread was then compared to the following: the control raw thread, composite-embedded thread without washing, and composite-embedded thread after washing. The resulting composite-embedded threads were analyzed using SEM.

#### 2.4.2 Antibacterial assay

The antibacterial activity of the TMNC coated on dental suturing thread was assessed using two methods: the well diffusion method and direct placement of the thread on agar plates to test against oral gram-positive and gram-negative bacteria [24]. The antibacterial assay for TMNC was conducted using Mueller-Hinton Agar (MHA) at a concentration of 3.8 % (w/v). To prepare this, MHA was diluted with double-distilled water, and additional agar was added. The mixture was then autoclaved at 121℃ for 15 psi for 15 min. This procedure was implemented to prevent any potential contamination. Following sterilization, the medium was poured into Petri dishes and allowed to solidify at room temperature. TMNC was then tested against two bacterial strains, *Escherichia coli* and *Staphylococcus aureus*, to evaluate its antibacterial properties. Initially, *E. coli* and *S. aureus* were cultured in nutrient broth at 37 °C for 24 h. The overnight culture was subsequently transferred into the fresh nutrient broth and incubated until the bacterial suspension reached a density of 0.5 McFarland turbidity standard, as determined by plate count. The chosen bacterial suspensions were evenly spread on MHA plates using sterile spreaders. Four wells of uniform size were created in the agar using 5 mm cork borer, and each well was filled with various concentrations of TMNC along with corresponding controls (Amikacin antibiotic (30 µg/disk) was used as a control purchased from Hi-Media). Subsequently, direct placement of the thread on agar plates was also done. The Petri dishes were incubated overnight at 37 °C for 24 h to measure the zone of inhibition (ZoI) in centimetres. All assays were conducted independently and in duplicate.

#### 2.4.3 MTT assay

The Human Oral Cancer cell line (KB) obtained from ATCC was cultured and utilized in the MTT assay to assess the cytotoxicity of TMNC. Cells were seeded at a density of 8 × 10^3^ per well in a 96-well plate and allowed to culture overnight in MEM medium. Following overnight incubation in MEM medium, the cells were treated with TMNC at dosages ranging from 0 to 15 μg/mL. After 24 h of further incubation, 20 μL of MTT reagent (5 mg/mL) was added to each well, and the plate was then kept in darkness for 4 h. Following the incubation period, an adequate amount of DMSO was added to each well, and the well was allowed to react for 15 min. The cell survival rate was determined by measuring the absorbance values at 590 nm using a microplate reader [25]. Cell viability was calculated as:

(1) $$Cell\;viability\;\left(\%\right)\;=\frac{\;Optical\;Density\;of\;test\;-\;Optical\;Density\;of\;blank}{Optical\;Density\;of\;control\;-\;Optical\;Density\;of\;blank\;}X100$$

#### 2.4.4 Antibiofilm activity of TMNC

Using polystyrene microtiter plates, the TMNC nanocomposite capacity to suppress the formation of biofilms in clinical isolates of *E. coli* and *S. aureus* was assessed at various TMNC ratios, as previously reported [26]. Shortly, sterile Luria Bertani broth (LB) was inoculated with pure colonies of *E. coli* and *S. aureus* isolates using aseptic handling. The mixture was then incubated for 24 h at 37 °C. Overnight culture was diluted in sterile LB to 1.5 × 10^8^ CFU/mL, or the 0.5 McFarland turbidity standard. The TMNC was prepared by diluting LB broth two-fold for the biofilm test, with no bacteria and nanocomposite as the negative control and without the use of nanocomposite as the positive control. In a 96-well plate, 10 µL of overnight culture was added to each well, and the plates were gently agitated before being placed in an incubator at 37 °C for 48 h. After the 48-h incubation period, the culture was collected, and the plates were washed three times with 200 µL phosphate-buffered saline (PBS) the wells were air dried Following the washes for 15 min, and stained with 100 µl/well of 1 % crystal violet solution left to incubate for 5 min. After staining, the wells were washed three times with copious amounts of distilled water. After washing, to dissolve the adhered dye, 30 % glacial acetic acid and 20 % acetone were added to each well, and the plate was further incubated for 15 min. Subsequently, the optical density (OD) at a wavelength of 570 nm was measured using a microplate reader, following the earlier reported method [27]. The percentage of biofilm inhibition was calculated using the following formula [28].

(2) $$\%ofInhibition=\frac{controlOD570nm-testODnm}{ControlOD570nm}X100$$

## 3 Results and discussion

### 3.1 Synthesis of nanocomposite consisting of Pb, Ag, and Cu

**Fig. 1:**
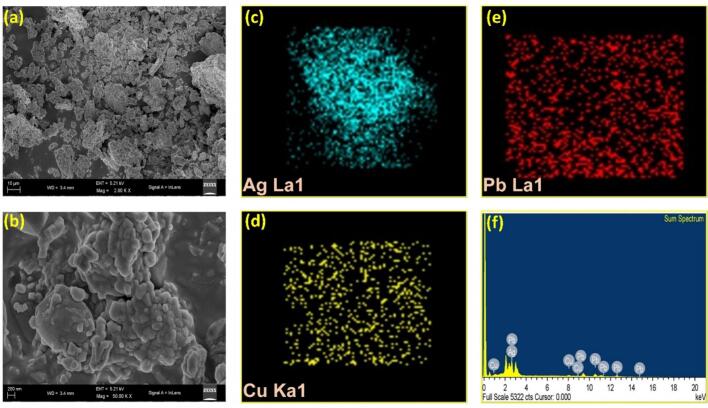
FESEM images and EDS mapping of metal composite: (a) 10 µm and (b) 200 µm magnifications, demonstrating the distribution of (c) Ag, (d) Cu, and (e) Pb, with (f) EDS spectrum of metal composite.

Using the facile hydrothermal technique, trimetallic Pb/Ag/Cu nanoparticles were successfully synthesized, exhibiting a highly porous structure. Fig. 1a and b present field emission scanning electron micrographs (FESEM) capturing the metal composite at two different magnifications: 10 µm and 200 nm. These images depict the morphology of the metal composite originating from three distinct metal precursors, revealing nanoparticle and nanocomposite sizes below 100 nm. Furthermore, the nanoparticle’s morphology exhibits various shapes, including rod-shaped, oval, spherical, and hexagonal structures.

In Fig. 1c, the EDS mapping illustrates the distribution of Ag, while Fig. 1d presents the distribution of Cu, and Fig. 1e displays the distribution of Pb. The results suggest that both Pb and Cu are uniformly dispersed, indicating good dispersion of the nanocomposites. The mapping of the Ag element reveals some concentrations of eutectic Ag in the alloy. Furthermore, Fig. 1f depicts the EDS mapping of the metal composite, highlighting the presence of the tri-metal, namely Ag, Pg, and Cu. The characterization of the metal composite using EDS confirms the presence of these three metals.

**Fig. 2:**
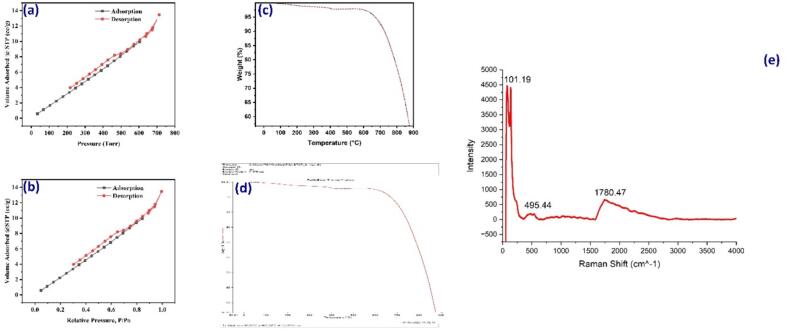
BET plots of the metal composite in panels (a) and (b), the TGA curves in panels (c) and (d), and the Raman spectrum of the metal composite in panel (e).

Fig. 2a and b present the nitrogen adsorption–desorption isotherms and BET plot of the mMOX at 77 K. The observed mean pore diameter, cumulative pore volume, and BET surface area of the mMOX were 8.41 nm, 0.0491 cm^3^/g, and 23.39 m^2^/g, respectively. The observed hysteresis loop in the graph indicates the mesoporous nature of the adsorbent, with the type IV isotherm classification following the IUPAC guidelines. Comparatively, the precursor utilized in nanoparticle synthesis exhibited a higher surface area than the calcinated samples. The calcination process resulted in a reduction in surface area compared to the precursor, primarily due to the original sample’s water absorption.

During BET measurements, the loss of water induces surface vacuolation, leading to an increase in surface area. Conversely, water is absorbed in the calcinated form of the sample. During calcination, both fluids and crystallization are eliminated, resulting in a reduction in surface area compared to the precursor. In solvothermal particle preparations, the incorporation of surfactants enhances the surface area and reduces particle size. However, when employing a solvothermal approach with MgO support and oleylamine as a reductive agent, there is a decrease in surface area compared to the coprecipitation method. This decline in surface area can be attributed to the increased concentration and decreased solvent volume within the autoclave [29]. The BET plots depicted in Fig. 2a and b for the metal nanocomposites illustrate a uniformly distributed specific surface area, suggesting a spherical composite structure [21]. Additionally, thermogravimetric experiments were conducted to evaluate the thermal stability of the synthesized metal composite. The experiments were conducted at a heating rate of 10 °C/min under a nitrogen atmosphere. Fig. 2c and d depict the thermal degradation pattern of the material. Analysis of the obtained DTG curve suggests that the metal composite remains stable below 700 °C. The observed weight loss of the material during the process is attributed to the evaporation of the absorbed moisture from the surroundings present on the surface of the metal composite. TGA analysis suggests that the synthesized metal composite exhibits thermally stability despite its small size. Furthermore, this metal composite shows promise as a potential photocatalyst.

The Raman spectra of oxide nanoparticle composites, synthesized using the arc-discharge method in distilled water (zone 2), exhibit various material phases, confirming previous findings. In the low-frequency range, below 1000 cm^−1^, prominent features include in-plane and out-of-plane CCC, CCN, and MgNC bending, along with MgN stretching and torsions. Notably, the shear mode C band (around 40 cm^−1^), arising from interlayer coupling, and the D’ band (approximately 1620 cm^−1^), activated by defects, are observed. Raman spectroscopy also facilitates the differentiation of various isotopes of carbon, with graphene exhibiting distinct Raman band peak positions for ^12^C and ^13^C isotopes due to their vibrational energy disparities. These observations provide valuable insights for studying relevant phenomena (Fig. 2e).

### 3.2 Analysis of Au/Pt/Cu trimetal using XPS

X-ray photoelectron spectroscopy (XPS) was conducted on the Au/Pt/Cu trimetallic nanocomposite to determine its surface chemical states and elemental composition. This analysis enabled the estimation of the valence state and binding energy of the elements present.

**Fig. 3:**
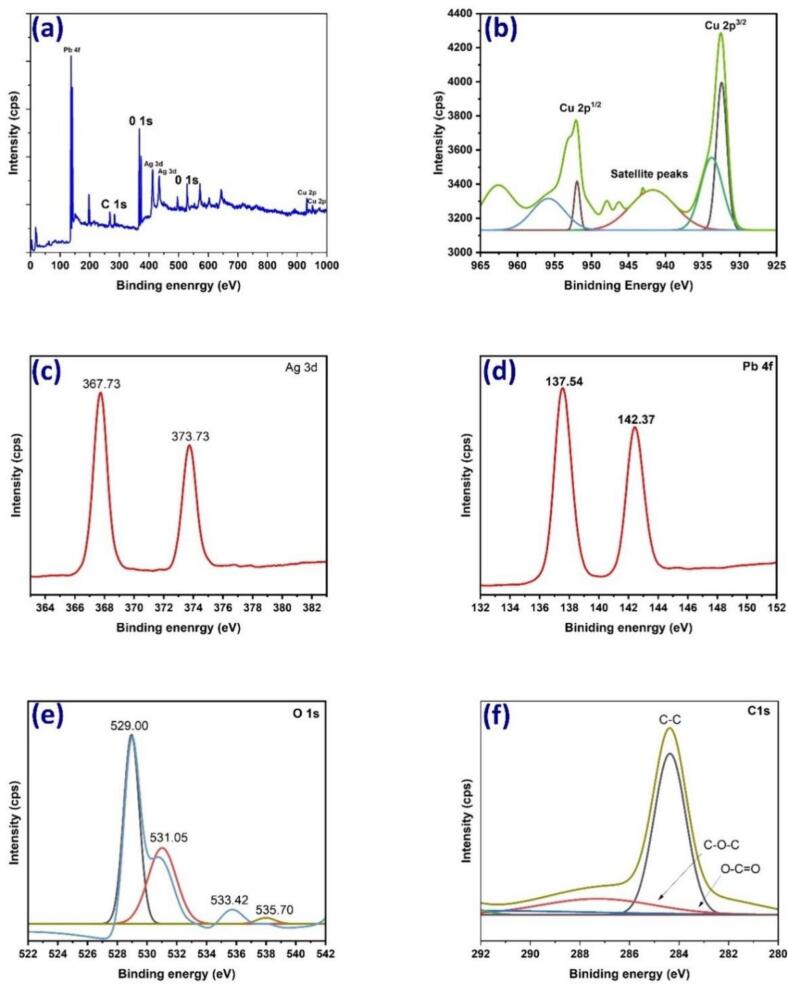
XPS spectrum of metal composite: spectra (a) through (f), with spectrum (d) specifically highlighting the XPS spectra of Ga2p and spectrum (e) presenting the XPS spectra of C1s.

Fig. 3a, b, c, and d exhibit the XPS spectra of CuO, AgO, and PbO nanoparticles, respectively. The binding energy (BE) was calibrated using the C 1 s electron peak at 283.5 eV. Specifically, the XPS BE values at 932.5 and 952.5 eV correspond to Cu 2p_3/2_ and Cu 2p_1/2_, respectively. Similarly, the XPS BE values at 367.73 eV, 373.73 eV, and 529 eV correspond to Ag, Pb, and O 1 s of CuO, AgO, and PbO nanoparticles, respectively. These results from the analysis are strongly supported by previously reported data. The XPS analysis indicated the absence of impurities such as Cu_2_O, Ag_2_O, and Pb_2_O in the sample, and no impurities like Cu (OH)_2_, Ag(OH)_2_, and Pb(OH)_2_ were detected. Furthermore, the reduction and stabilization process of the metal composite, involving the reduction and binding of the trimetal Ag, Cu, and Pb, was well-established in the XPS analysis.

### 3.3 FTIR spectrum analysis

**Fig. 4:**
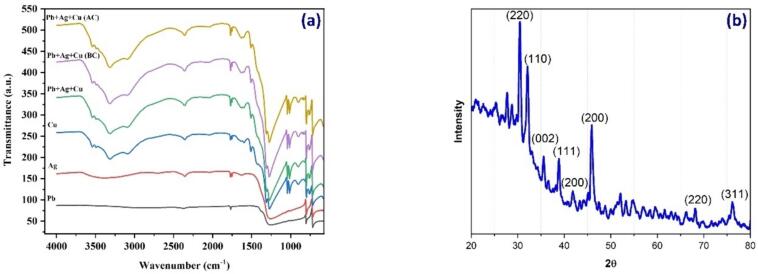
(a) FTIR spectrum of the metal composite compared to individual metals, (b) XRD spectrum of the metal composite.

Fig. 4a presents the FTIR spectrum of a metal composite, displaying both an individual metal and a trimetallic metal composite. This spectrum illustrates the characteristic vibrations between the bonds of atoms in nanoparticles. The absorption peaks observed in the FTIR spectrum assist in identifying the functional groups present in the nanoparticles. As each type of nanoparticle exhibits a unique combination of atoms, the number of functional groups present is determined by the size of the peaks in the spectrum.

### 3.4 X-ray diffraction analysis

The amorphous nature of the synthesized nano metal composite was confirmed by observing its peak (Fig. 4b). Furthermore, the formation of the metal composite was validated through XRD analysis, which revealed the presence of monoclinic crystallites without any impurities. The presence of crystallites was indicated by the observed planes (2 2 0), (1 1 0), (0 0 2), (1 1 1), (2 0 0), (2 0 0), (2 2 0), and (3 1 1) in the XRD analysis. Notably, significant 2θ values appeared at 32.5, 33.5, 35, 38, 42.2, 47.5, 69, and 76.5, corresponding to the (2 2 0), (1 1 0), (0 0 2), (1 1 1), (2 0 0), (2 0 0), (2 2 0), and (3 1 1) planes, respectively.

### 3.5 SEM image of dental suturing thread coated with metal composite

**Fig. 5:**
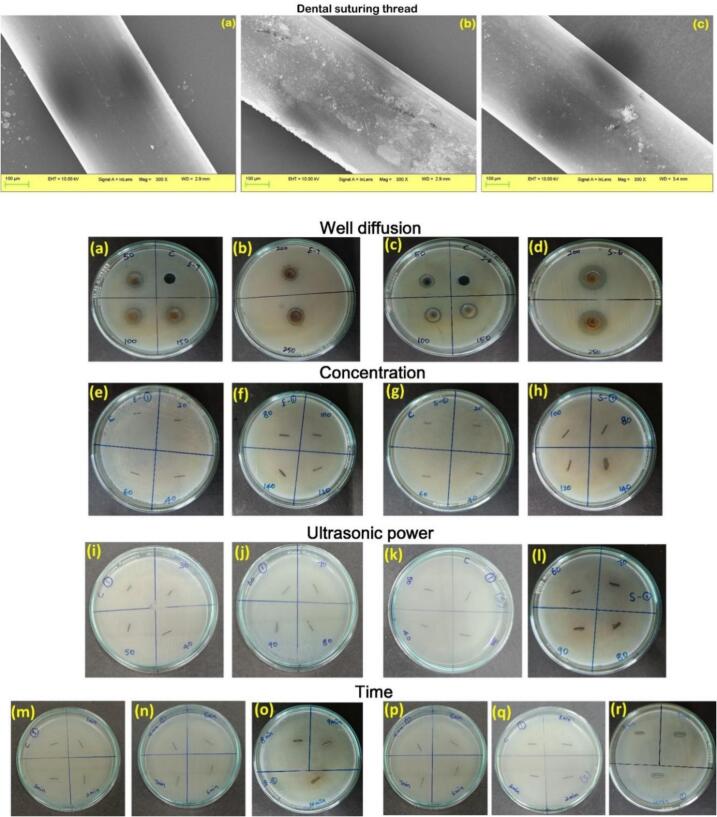
Illustration of metal composite-coated dental suturing thread: SEM images (a) for the control, (b) for the metal composite-coated, and (c) for the metal composite-coated thread after washing. Antibacterial activity against *E. coli* and *S. aureus*: results from the well diffusion method (a-d). Antibacterial activity of the composite-coated dental suturing thread: against *E. coli* and *S. aureus* with different concentrations of the composite coating under ultrasonic power (e-h), under various ultrasonic powers (i-l), and coating of dental suturing thread under different time durations (m-r).

The dental suture material, following coating with the synthesized metal composite, was examined under the scanning electron microscope (SEM) (Fig. 5a control, 5b metal composite-coated, and 5c metal composite-coated thread after washing). The results revealed the presence of nanoparticles in the dental suture, demonstrating the efficiency of the trimetallic composite in enhancing the dental suture material. Moreover, subsequent examination under SEM after washing confirmed the continued presence of nanoparticles in the image, indicating the sustained effectiveness of the material even after washing.

#### 3.5.1 Antimicrobial effects of metal composites

The antibacterial effectiveness of the different concentrations of the metal composite was evaluated against both gram-positive (*E. coli*) and gram-negative (*S. aureus*) bacteria (Fig. 5a-d). The study examined the antibacterial activity of the synthesized metal composite at various concentrations (50,100, 150, 200, 250 µg/mL). Specifically, the antibacterial efficacy of a dental suture coated with the metal composite against both gram-negative and gram-positive bacteria was assessed at different concentrations. Composite-coated dental suturing thread: against *E. coli* and *S. aureus* with different concentrations of the composite coating under ultrasonic power (Fig. 5e-h), under various ultrasonic powers (Fig. 5i-l), and coating of dental suturing thread under different time durations (Fig. 5m-r). The findings revealed that as the concentration increased, the antibacterial effectiveness also increased, leading to a larger zone of inhibition. The experiment assessed the zone of inhibition at concentrations of 20, 40, 60, 80, 100, and 140 µg/mL, demonstrating a corresponding increase in the zone of inhibition with higher concentrations. Furthermore, higher ultrasonic power contributed to enhanced antibacterial activity, as evidenced by an augmented zone of inhibition following 10 min of ultrasonic power exposure. Moreover, a concentration of 90 µg/mL also led to an amplified zone of inhibition. These results suggest that the dental suture material incorporating the metal composite effectively inhibits bacterial growth, highlighting its potential as an antibacterial agent.

SEM images comparing the control, coated, and washed metal composite-coated dental suturing thread were analyzed [32]. The SEM image of the dental suture material revealed the presence of nanoparticles in the coated material, indicating successful coating with the metal composite. However, upon examination of the washed material under SEM, no nanoparticles were detected, suggesting that the washing process effectively removed any loosely attached nanoparticles from the surface of the dental suture thread.

#### 3.5.2 Metal composite using MTT assay

**Fig. 6:**
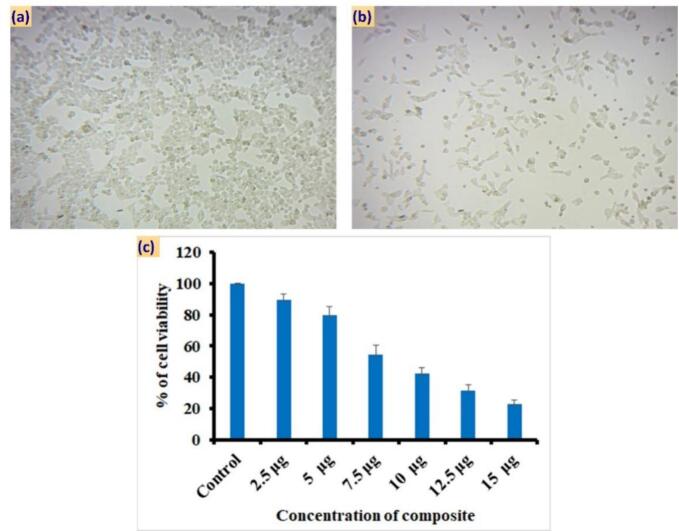
MTT assay of metal composite against KB cell line.

Cell viability of the KB cell line was evaluated at various concentrations of the metal composite (Fig. 6). Interestingly, viability was observed to be satisfactory at both higher and lower concentrations, indicating the compatibility of the cells with these extremes. At a low concentration of 2.5 µg, biofilm growth was 96 %, while at 15 µg, it was only 27 %. However, viability was found to be lower at intermediate concentrations, suggesting a potential cytotoxic effect of the metal composite within this concentration range. In addition to the traditional endpoint viability assays, *in-situ* monitoring of the KB cell line was conducted during exposure to the synthesized metal composite. This real-time monitoring allowed for dynamic observation of cellular responses and provided insights into the temporal effects of the metal composite on cell viability and behaviour [31]. In this invitro cell culture study, we observed a concentration-dependent cytotoxic effect of TMNC on oral squamous cancer cells. The observed cytotoxicity was significant at all sequential doses compared to control (p < 0.0001).

**Fig. 7:**
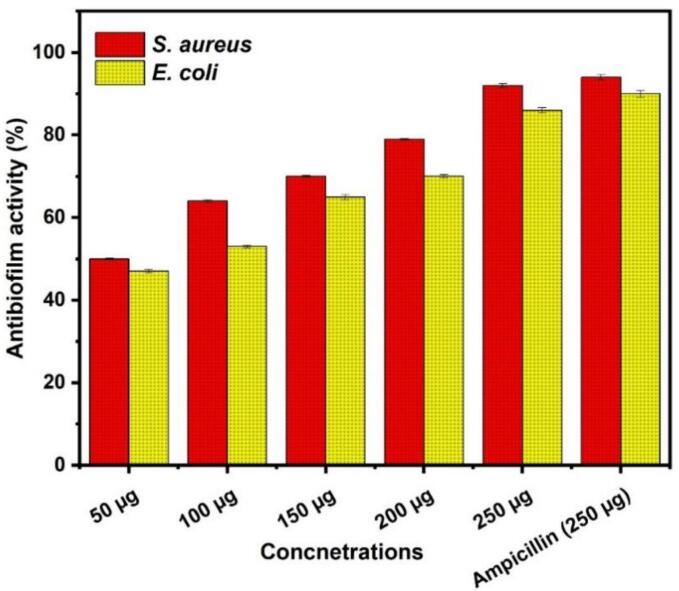
Antibiofilm activity of metal composite at different concentrations.

The efficacy of the TMNC nanocomposite in suppressing the production of biofilms by *S. aureus* and *E. coli* was examined using crystal violet staining, as seen in Fig. 7. The graphs (Fig. 7) demonstrated how, at varying percentages of inhibition, every ratio of TMNC concentration under investigation suppressed the activity of biofilm development. Greater TMNC concentrations prevented the formation of biofilms from *S. aureus* and *E. coli* at 85 % and 83 %, respectively. The percentage inhibition increased along with the TMNC nanoparticle concentration. The percentage of biofilm inhibition of the nanocomposite for both bacteria rose with an increase in the TMNC ratio. This is a result of TMNC’s antibiofilm function. The concentration of TMNC increases the biofilm inhibition and also increases for both bacteria. This was following an earlier report by Garza-Cervantes et al. [30] on the gradual decrease in biofilm formation when the concentration of composite nanoparticles increased.

## 5 Conclusion

The innovative development of dental suture materials incorporating TMNC represents a significant advancement in medical textiles. Its application in dental sutures reduces the risk of infections that may compromise the patient’s recovery and oral health in dental surgery. This study investigated the enhanced antibiofilm and antibacterial activities of these materials. Utilising the MTT assay, the efficacy of the metal composite against the KB cell line is highlighted, presenting its potential in cellular-level applications. Furthermore, evaluation of the antibiofilm activity demonstrated the effectiveness of the synthesised metal composite in combating biofilm formation, a crucial aspect in preventing infections associated with medical procedures. The combined antibacterial and antibiofilm properties present promising prospects for medical applications, particularly in dental procedures where infection prevention is critical. Moreover, *in-situ* monitoring of the local respiratory activity of human nasopharyngeal epithelial cancer (KB) cells during exposure to the synthesized metal nanocomposite revealed its significant influence on cellular respiration processes. The discovery of cell toxicity towards potential disease-causing cells suggests the utility of the metal composite in treating conditions such as cancer and autoimmune disorders. The integration of nanotechnology and nanomedicine offers a more targeted and effective approach to medical textile development, including dental sutures. This study highlights the potential scope of metal composites to revolutionize medical textiles, paving the way for advanced applications in infection prevention, cellular therapy, and disease treatment.

## CRediT authorship contribution statement

**Kanagasabapathy Sivasubramanian:** Data curation. **Yuvaraj Tamilselvi:** Formal analysis. **Palanivel Velmurugan:** Conceptualization. **Fatimah Oleyan Al-Otibi:** Funding acquisition. **Raedah Ibrahim Alharbi:** Investigation. **Vinayagam Mohanavel:** Methodology. **Sivakumar Manickam:** Project administration. **Jeyanthi Rebecca L.:** Software. **Basavaraj Rudragouda Patil:** Resources.

## Declaration of competing interest

The authors declare that they have no known competing financial interests or personal relationships that could have appeared to influence the work reported in this paper.
